# An Adaptive Role for DNA Double-Strand Breaks in Hippocampus-Dependent Learning and Memory

**DOI:** 10.3390/ijms23158352

**Published:** 2022-07-28

**Authors:** Sydney Weber Boutros, Vivek K. Unni, Jacob Raber

**Affiliations:** 1 Department of Behavioral Neuroscience, Oregon Health & Science University, Portland, OR 97239, USA; webesy@ohsu.edu; 2 Department of Neurology, Oregon Health & Science University, Portland, OR 97239, USA; unni@ohsu.edu; 3 Jungers Center for Neurosciences Research, Oregon Health & Science University, Portland, OR 97239, USA; 4 Oregon Health & Science University Parkinson Center, Portland, OR 97239, USA; 5 Department of Radiation Medicine, Oregon Health & Science University, Portland, OR 97239, USA; 6 Division of Neuroscience, Oregon National Primate Research Center, Beaverton, OR 97006, USA

**Keywords:** DNA damage, cognition, double-strand brakes, hippocampus, aging, etoposide, amifostine

## Abstract

DNA double-strand breaks (DSBs), classified as the most harmful type of DNA damage based on the complexity of repair, lead to apoptosis or tumorigenesis. In aging, DNA damage increases and DNA repair decreases. This is exacerbated in disease, as post-mortem tissue from patients diagnosed with mild cognitive impairment (MCI) or Alzheimer’s disease (AD) show increased DSBs. A novel role for DSBs in immediate early gene (IEG) expression, learning, and memory has been suggested. Inducing neuronal activity leads to increases in DSBs and upregulation of IEGs, while increasing DSBs and inhibiting DSB repair impairs long-term memory and alters IEG expression. Consistent with this pattern, mice carrying dominant AD mutations have increased baseline DSBs, and impaired DSB repair is observed. These data suggest an adaptive role for DSBs in the central nervous system and dysregulation of DSBs and/or repair might drive age-related cognitive decline (ACD), MCI, and AD. In this review, we discuss the adaptive role of DSBs in hippocampus-dependent learning, memory, and IEG expression. We summarize IEGs, the history of DSBs, and DSBs in synaptic plasticity, aging, and AD. DSBs likely have adaptive functions in the brain, and even subtle alterations in their formation and repair could alter IEGs, learning, and memory.

## 1. Introduction

The mechanisms underlying neuronal signaling in the hippocampus have been explored extensively for decades. Dr. Brenda Milner’s work with Henry Gustav Moliason (H.M.) in the late 1950s was key in identifying the importance of the hippocampus for explicit (requiring conscious effort) learning and memory [1]. H.M. developed life-long, explicit anterograde amnesia following a bilateral temporal lobectomy (which removed most of his hippocampi) to address his severe epilepsy. Subsequently, research into the hippocampus identified it as an important structure for spatial encoding, navigation, and memory consolidation [2]. The underlying cellular mechanisms involve the expression of immediate early genes (IEGs) and might relate to DNA double-strand breaks (DSBs). Recent investigations have pointed to DSBs as a mechanism leading to the rapid expression of IEGs following stimulation: activation of central nervous system (CNS) cells via N-methyl-D-aspartate receptors (NMDARs), α-amino-3-hydroxy-5-methyl-4-isoxazolepropionic acid receptors (AMPARs), electrical currents, serum, heat shock, and behavioral paradigms have all led to transient increases in DSB markers and increased IEG expression, suggesting a so-called “adaptive” role for DSBs. In this review, we provide a brief history of DSBs, novel research suggesting a role of DSBs in IEG expression, and evidence from healthy aging and neurodegeneration research that contributes to the argument for an adaptive role of DSBs in the CNS.

## 2. Immediate Early Genes in Synaptic Plasticity, Learning, and Memory

The category “immediate early genes” (IEGs) comprises genes encoding a family of proteins linked to synaptic plasticity, learning, and memory [3,4]. IEGs are induced by numerous stimuli—from behavioral experiences to drugs of abuse. IEGs are expressed at very low baseline levels. Within minutes of stimulation, IEGs are transiently increased. IEGs do not require de novo protein synthesis for their transcription, as inhibition of protein synthesis does not impair IEG expression [5,6,7,8]. The mechanisms behind this rapid induction of IEG expression are still unclear, though components such as MAPK/ERK, CREB, and calcium are known to be necessary for IEG induction [4,9,10]. Expression of IEGs is cleared relatively quickly, returning to baseline levels within 2–3 h following stimulation [11]. Because of this, IEGs are widely used as markers of neuronal activation in the central nervous system [4,5,11,12,13].

The functions of IEGs range from initiating transcription to direct effects on synaptic maintenance. This has led to their sub-classification into “effector” IEGs—genes that directly affect cellular function—or “regulatory transcription factors” (RTFs)—genes that affect cellular function indirectly by regulating expression of other genes [14,15,16,17]. It is estimated that the majority of IEGs are effectors, while ~15 are RTFs [14,16]. A select few IEGs linked to DSBs are summarized in this section (Table 1).

*Fos and Jun.* The first description of the proto-oncogene *c-fos* in the brain was following the induction of seizures [18]. mRNA levels of c-Fos peak around 60 min after seizure induction, while protein levels peak around 90 min. Levels of mRNA transiently go below baseline 3 h post-stimulus and then return to baseline levels at 16 h. Around the same time, *c-Jun* was identified as a component of the activator protein 1 (AP-1) transcription factor [19]. c-Fos and c-Jun form a heterodimer to create the AP-1 complex; once formed, AP-1 subsequently acts as a transcription factor, indirectly contributing to synaptic plasticity by inducing the transcription of genes needed for synaptic growth [20].

There is a wealth of literature from the last 40 years about the role of *fos, jun,* and the AP-1 complex in learning and memory. *c-fos* is upregulated in anatomically relevant regions following fear conditioning, including the mammillary bodies and anterior thalamus [21] and in the dorsal CA1 hippocampal region [22]. Moreover, the inhibition of c-Fos in the CA1 region impairs contextual recall [23].

Other members of the *fos* and *jun* families have been identified, such as FosB, JunB, and JunD [24,25]. Less is known about them, though FosB and a splice variant, ΔFosB, appear to persist as a more long-term molecular switch [24,26,27,28]. JunB is also upregulated in the hippocampus after contextual fear conditioning [22], suggesting a role in contextual encoding. Additionally, the binding of JunD to the AP-1 complex is associated with fear extinction and decreased c-Fos in the dorsal hippocampus [29].

*Arc/Agr3.1*. In contrast to *fos* and *jun*, the activity-regulated cytoskeletal-associated gene (*Arc*, otherwise known as *Agr3.1*) is an example of an “effector” IEG [16,30,31]. It is a highly regulated protein primarily expressed in neurons. Following activation, it is rapidly targeted to activated synapses, where it increases the number of thin spines and decreases the density of AMPA receptors [32]. *Arc* knock out mice display intact short-term memory [33], but inhibition or lack of Arc impairs late-LTP [34] and long-term memory consolidation [33]. Inhibiting Arc after fear conditioning also impairs hippocampus-dependent contextual fear memory [35], highlighting the importance of Arc in memory maintenance. *Arc* expression in the dorsal hippocampus (compared to the ventral hippocampus) appears to be especially important for spatial memory [36].

*Npas4.* Neuronal PAS domain protein 4 (*Npas4*) is another RTF. *Npas4* maintains the balance between excitation and inhibition: one of its major downstream effects is increasing inhibitory synapses on cell bodies, while decreasing inhibitory synapses on apical dendrites in the CA1 [37]. This appears to be modulated by the downstream activation of brain derived neurotrophic factor (BDNF) [13]. The accurate maintenance of inhibitory signaling is especially crucial during development and adolescence. Npas4 is important for inhibitory synapse development in the prefrontal cortex during adolescence [38], implicating *Npas4* dysregulation in disorders such as schizophrenia [39]. The deletion of *Npas4* with a Cre construct in the CA3 of mice impairs contextual fear memory and inhibits mossy fiber connections [40]. Moreover, increased *Npas4* expression suppresses fear memory in a post-traumatic stress disorder (PTSD) mouse model [41], further highlighting the importance of inhibitory signaling related to psychological disorders.

*Other IEGs.* Other IEGs include *Egr1/zif268*, *Homer1*, and *Cyr61*. *Egr1*—also known as *zif268*—is expressed following contextual fear conditioning [42] and induced by LTP [43]. *Egr1* appears to be both an effector and an RTF: its exact transcriptional targets are unknown, but it binds to DNA and promotes transcription, as well as works directly in response to cellular damage [44]. *Homer1* is an effector IEG that mediates the binding between mGluRs and IP3 receptors, thereby negatively regulating excitatory synapses [13]. *Homer1a* is upregulated in the CA1, CA3, and dentate gyrus following exposure to a novel environment, contextual fear conditioning, and contextual memory test [45]. Lastly, *Cyr61* plays an essential role in dendritic growth in the hippocampus [46].

**Table 1:** Selection of IEGs and their known primary function in synaptic plasticity.

Name	Classification	Primary Function	References
*c-Fos*	RTF	Binds with cJun to create the AP1 complex, thereby promoting transcription	[20]
*c-Jun*	RTF	Binds with cFos to create the AP1 complex, thereby promoting transcription	[20]
*Arc/Agr3.1*	Effector	Involved in endocytosis of AMPA receptors and increasing thin spines	[13]
*Npas4*	RTF	Mediates the balance between inhibitory and excitatory signals, notably by controlling inhibitory synapse growth	[13]
*Egr1/zif268*	RTF and Effector	Transcription factor; important in cell survival, differentiation, and death, especially after injury	[42,43]
*Homer1a*	Effector	Negative regulation of excitatory synapses via mediating the binding between mGluRs and IP3 receptors	[13]
*Cyr61*	Effector	Promotes adhesion of endothelial cells and aids in DNA synthesis; regulates dendritic growth	[46]

## 3. DNA Double-Strand Breaks: A Dangerous and Complex History

Maintaining DNA integrity is critical for faithful gene expression. Yet, DNA is susceptible to many forms of damage during normal cellular functioning: it is estimated that up to tens of thousands of DNA-damaging events happen daily per cell [47]. These include base-pair mismatch, which can occur during the DNA replication process; oxidative base damage; single-stranded breaks (SSBs), often induced by ionizing radiation or reactive oxygen species; and double-stranded breaks (DSBs) [48]. All types of DNA damage have the potential to lead to negative consequences, such as apoptosis or tumorigenesis, but DSBs are considered the most cytotoxic [49]. When unrepaired, they serve as a signal for cell death [50,51,52].

DSBs can result from chemical toxins (such as cisplatin, bleomycin, and etoposide) and ionizing radiation (IR) [51]. They can also be harnessed for therapeutic effects in chemotherapy and radiation therapy (RT) to treat cancers. Chemical- and RT-induced DSBs kill tumorigenic cells and eliminate tumors; however, side effects, including those involving injury to healthy bystander cells, are common and debilitating [53]. IR often leads to formation of many SSBs; when SSBs are close together and present in opposite DNA strands they produce DSBs, often with “dirty ends” (single-stranded overhangs) that lead to deletion of nucleotides during the DSB repair process [54].

Yet, DSBs are not always pathological. They play an important, adaptive role in the immune system, including the generation of antibody diversity during V(D)J recombination and class-switching of immunoglobulins [55]. DSBs are also seen in cell division during the S/G2 phases [56]. The fine line between damage-induced/pathological versus physiologically relevant/adaptive DSBs is maintained by faithful and efficient DSB repair. In a healthy functional cell, DSBs that form during mitosis are repaired via homologous recombination (HR), where breaks are mended using a sister chromatid strand to faithfully restore genetic information that may have been lost when the break formed [51]. DSBs induced by damage, or DSBs in non-dividing cells, are primarily repaired by non-homologous end joining (NHEJ), whereby a series involving the processing and ligation of the two DNA ends. This process is highly error-prone, offering the opportunity for insertions and deletions to form [49,50].

Rapidly upon DNA breaking, histone variant H2Ax is phosphorylated at serine-139, forming γH2Ax, which occurs near the site of the break [57]. γH2Ax appears to play an important role in initiating DSB repair, by increasing accessibility to the broken DNA and recruiting other proteins important for the repair process [57]. One of these proteins—p53 binding protein 1 (53BP1)—is a marker for repair by the NHEJ pathway [58]. Due to the rapid focal accumulation of these two proteins at DSB sites, they are often used to identify the anatomic and genomic location of breaks (γH2Ax) and active repair (53BP1).

The adaptive roles of DSBs have been primarily described in peripheral, dividing cells. Yet, the central nervous system (CNS) is comprised of post-mitotic cells. As a result, DSBs in the CNS have been characterized as harmful as a result. DSBs in the hippocampus specifically have been identified throughout the lifespan as harmful for learning, memory, and general survival. Mice irradiated at prenatal days 3, 10, or 21 had persisting γH2Ax in the dentate gyrus of the hippocampus until 15 months of age and decreased survival [59]. Repeated exposure to fractionated low-dose radiation (0.1 Gy) in juvenile and adult mice increased 53BP1 in the dentate gyrus and decreased neurogenesis, suggesting long-term increased DNA damage and cell death [60].

## 4. A Role for DSBs in IEG Expression, Learning, and Memory

In a sudden shift, recent reports suggest that DSBs may also have an adaptive role in physiological brain function. Increasing evidence supports a connection between DSBs and IEGs, potentially offering an explanation for this rapid expression upon stimulation (Table 2).

**Table 2:** Summary of findings related to an adaptive role of DSBs in IEG expression.

Reference	Year	System	Sex	Age	Stimulation	Main Findings	IEGs Upregulated	IEGs Unchanged
Crowe et al. [61]	2006	Primary cortical rat neurons	Not reported	Not reported	AMPA, NMDA, Electrical pulse	Sub-toxic stimulation of ionotropic glutamate receptors resulted in γH2Ax formation (NDMA increased within 10 min, AMPA increased within 30 min)	n/a	n/a
Madabhushi et al. [62]	2015	Primary hippocampal mouse neurons Wild-type mice	Not reported	Not reported	Potassium chloride, bicucullin, NMDA, etoposide Fear conditioning	Physiological stimulation induces DSBs on transcriptional start sites that leads to upregulation of a sub-set of genes, mostly IEGs	Fos, FosB, Nr4a1, Npas4	n/a
Bunch et al. [63]	2015	HEK239 Cells	n/a	n/a	Heat Shock Serum	DSBs occur downstream of TSS, leading to transcriptional elongation.	Egr1, Fos, Jun, Myc	n/a
Suberbielle et al. [64]	2013	Wild-type/APP-PS1 mice	Males and Females	4–7 months	Exposure to novel environment Visual stimulation	Transient increase in γH2Ax foci in relevant brain regions; high baseline levels of γH2Ax in APP/PS1 mice and elevated levels at 24 h compared to WT mice.	n/a	n/a
Li et al. [65]	2019	Wild-type mice	Males	2 months	Trace fear conditioning	Inducing DSBs with etoposide prior to trace fear conditioning led to prolonged increase of IEG expression and impaired memory	Arc, cFos, Cyr6, Npas4	n/a
Boutros et al. [66]	2022	Wild-type mice	Males and Females	3–4 months	Fear conditioning +/− systemic amifostine or etoposide	Increase contextual fear memory in males that received amifostine; decreased contextual and cued fear memory in females that received etoposide. Sex-dependent changes in hippocampal ΔFosB after etoposide.	ΔFosB	cFos
Navabpour et al. [67]	2020	Sprague Dawley rats	Males	2 months	Fear reconsolidation	Increased DSBs in promoter region of Npas4 following fear memory test; impaired fear retention following inhibition of topoisomerase IIβ	Npas4	cFos
Kugelman et al. [68]	2016	Wild-type mice	Males	1.5 months	Whole-body gamma irradiation	Whole-body gamma irradiation after fear training led to increased fear expression but decreased cFos in GABA cells in the infralimbic cortex	cFos	n/a
Stott et al. [69]	2021	Wild-type mice	Males	4 months	Contextual fear conditioning Glucocorticoids	Increased DSBs in neurons and glial following contextual fear conditioning Glial cells have an increase DSBs in response to corticosterone	Egr1, Egr3, Junb, Npas4, Nr4a1	n/a
Bellesi et al. [70]	2016	Drosophila Wild-type mice	Males and Females	3 months	Exposure to novel environment Whole-body gamma irradiation	Increased markers of DSB repair during sleep; impaired DSB repair when sleep is prevented	n/a	n/a

An early study identified that sub-toxic stimulation of ionotropic glutamate receptors in cultured rat neurons result in γH2Ax formation and cell survival [61]. Transient increases in γH2Ax and MRE11—the DNA damage response complex needed for initiation of DSB repair—are observed following the activation of NMDARs (15 μM) or AMPARs (25 μM). NMDAR activation leads to peak γH2Ax levels 10 min later, while AMPAR activation leads to peak γH2Ax signal 30 min later. Vitamin E and the calcium-chelator BAPTA attenuate this increase, though not to baseline levels, indicating that while calcium plays a role, DSBs formed following subtoxic glutamatergic stimulation are not entirely calcium-dependent.

DSBs were also shown to be induced by stimulating cultured hippocampal mouse neurons. These breaks lead to increased gene expression in a specific subset of genes, comprising of genes related to synaptic growth and maintenance (including the IEGs *Fos, Npas4,* and *Egr1*), and occur at promoter regions [62]. Inhibiting topoisomerase-II β (topoIIβ), which cuts both backbones of DNA simultaneously to [71], decreases formation of γH2Ax and IEG expression, indicating that DSBs induced by neuronal activation are dependent on topoIIβ. Activity-induced DSBs may remove physical constraints preventing promoter and enhancer regions from interacting, allowing rapid transcription to occur [72]. Moreover, mice trained in a contextual fear conditioning test show an increase in γH2Ax foci in the hippocampus, though IEG expression was not assessed [62].

**Figure 1:**
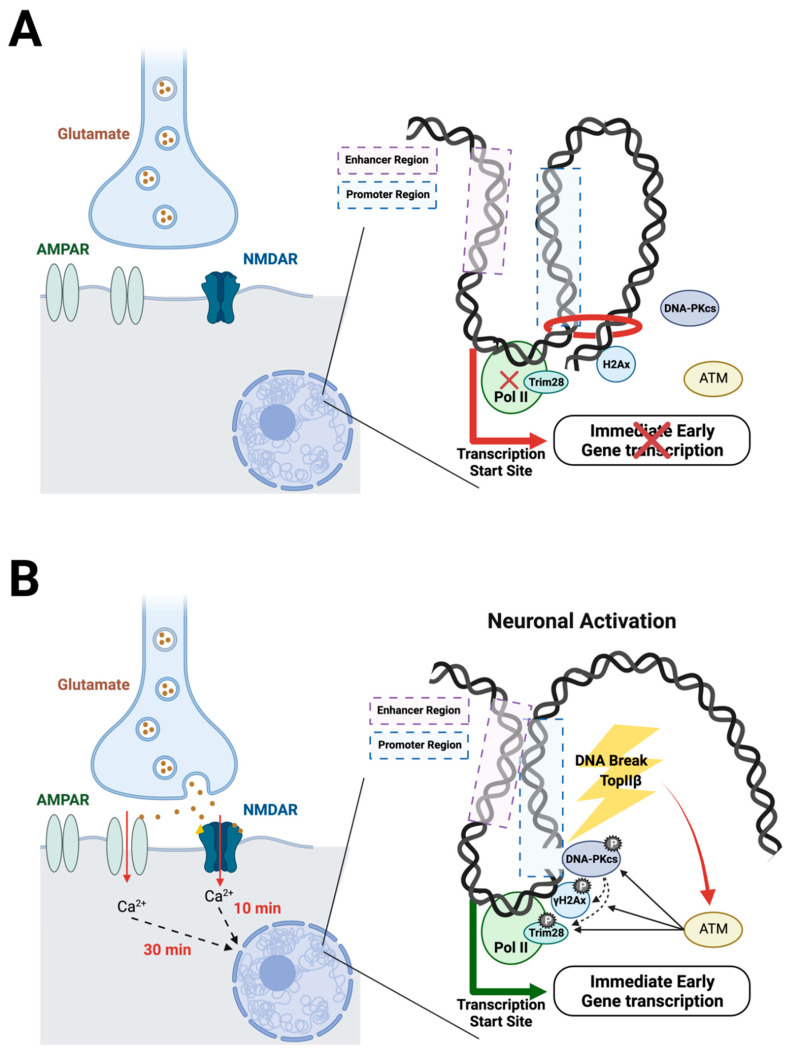
Suggested mechanims of adaptive DSBs. (**A**) At baseline, transcription is held in pause. Trim28 holds RNA polymerase II (Pol II) in pause, and topological factors prevent the enhancer and promoter regions from interacting. (**B**) Neuronal activation occurs with the binding of glutamate to NMDARs and AMPARs, allowing an influx of calcium that initiates a signal cascade into the nucleus. Following this cascade, topoisomerase II β initiates a DSB downstream of the transcription start site. The DSB activates ATM, which induces phosphorylation of DNA-PKcs, Trim28, and H2Ax. Trim28 phosphorylation releases Pol II, which then becomes active to induce transcription of IEGs. Additionally, the DSB releases topological constraints, allowing the enhancer and promoter regions to interact.

Another report showed evidence that DNA breaks are involved in transcriptional elongation [63]. Using HEK293 cells, DSBs induced by serum are found downstream of transcriptional start sites (TSS) of the IEGs *Egr1*, *Jun*, *Fos*, and *Myc*. These IEGs are also upregulated, supporting that the DSBs induced by physiological activation are involved in gene expression. The ATM serine/threonine kinase (ATM) and DNA-dependent protein kinases (DNA-PKcs)—both known responders to DNA damage—phosphorylate Trim28, which holds RNA polymerase II (PolII) in pause. Once phosphorylated, Trim28 releases the pause on PolII, allowing transcription to begin. Again, the data indicate that topoIIβ is important in these DSBs [62,63]. Together, these studies suggest that neuronal activation induces topoIIβ-mediated DSBs, which in turn activate ATM and DNA-PKcs that phosphorylate Trim28 and H2Ax, which release the pause on PolII and begin the DSB repair process, ultimately resulting in the transcription of a subset of genes (Figure 1).

Moving to in vivo models, a transient increase in γH2Ax was observed in the brains of C57BL/6J wild-type (WT) mice following exposure to a novel environment [64]. γH2Ax levels were low at baseline, returned to these baseline levels in 24 h, and were seen exclusively in brain regions important for contextual encoding, such as the parietal cortex and dentate gyrus. Similarly, visual stimulation resulted in an increase in γH2Ax in the corresponding hemisphere of the visual cortex, but not the opposite hemisphere [64]. This region-specific change in γH2Ax levels suggest that typical, external stimuli trigger sub-toxic DSBs.

### 4.1. Importance of DSB Repair

These studies subsequently spurred interest in how interfering with typical DSB formation and/or repair might affect learning and memory. If DSBs are involved in IEG expression, then manipulating DSBs and/or DSB repair would alter gene transcription and ultimately cause long-term memory disruptions [73]. One target to interfere with DSB repair is the Gadd45 family of proteins, which is involved in DNA repair and de-methylation [74]. Inhibition of Gadd45γ in the prelimbic area during trace fear conditioning alters IEG expression and decreases long-term memory [65]. While the first wave of IEG expression is not altered by the Gadd45γ inhibitor, the second peak of IEG signaling (specifically *Arc, cFos, Npas4,* and *Cyr61*) at 5 h post-test is affected. This suggests that timely DSB repair is important in the regulation of IEG expression and the learning process.

### 4.2. Importance of DSB Timing

To manipulate DSB formation, etoposide (also known as Vepesid, Etopophos, or Toposar) is a useful tool. Etoposide induces DSBs and is used during chemotherapy to increase apoptosis in tumors [75]. Etoposide increases DSBs by interfering with the complex of covalently-bound topoIIβ to cleaved DNA [76]. In the clinic, major side effects of etoposide include a host of gastrointestinal disturbances, as well as extreme fatigue and weakness [77]. Etoposide has a lower half-life in females, though clearance is similar between the sexes [78]. The use of etoposide in the prelimbic area of male mice impairs expression of *Arc, cFos, Npas4*, and *Cyr61* at 5 h post-test; mice that receive etoposide infusions also display decreased freezing in response to the conditioned stimulus [65]. Systemic administration of 35 mg/kg of etoposide just after training in fear conditioning impairs long-term contextual (hippocampus-dependent) fear memory in female mice and cued (hippocampus-independent) fear memory in both male and female mice [66]. Hippocampal cFos and ΔFosB are unchanged immediately after drug administration, but long-term (2 weeks later) hippocampal ΔFosB is increased in males that receive etoposide just after training. Moreover, etoposide applied to MCF7 (breast cancer) cells in vitro leads to the upregulation of gene transcription, mostly related to gene activity, protein binding, and neurogenesis [79]. These studies indicate that precise DSB formation is also important for in-tact IEG expression and long-term learning and memory.

Looking at another method, the induction of DNA damage with low-dose radiation (0.1 Gy at 20 fractions) affects IEGs and phospho-cAMP Response Element-Binding Protein (pCREB) in differing directions at distinct times post-radiation [60]. pCREB, an important transcription factor for IEGs, is decreased 72 h and 1-month post-radiation, but increased 3 months later. Arc and BDNF are also increased 3 months after radiation, while markers of neurogenesis are decreased. Proper regulation of DSBs might contribute to synaptic growth, though DSBs likely need to occur at the correct time and the correct place to be adaptive. The DSBs induced by etoposide or radiation are likely occurring out of time and place, and therefore leading to disrupted IEG expression, learning, and memory.

Conversely, amifostine (also known as Ethyol) protects non-cancerous cells from DSBs during RT and is commonly co-administered during treatment [80]. Amifostine, metabolized into the active agent WR-1065, scavenges free radicals and appears to increase the speed of DSB repair, preferentially protecting non-tumor tissue [80,81]. Side effects range from gastrointestinal disturbances to severe skin conditions, with a high rate of adverse side effects leading to discontinuation of use, especially in head and neck cancers [82]. In cancer patients, women clear plasma amifostine faster than men [83]. Systemic administration of amifostine (214 mg/kg) before gamma radiation rescues object recognition and restores hippocampal neurogenesis in male mice [84]. Amifostine appears to have long-term protective effects: a single systemic administration of 107 or 214 mg/kg prior to simulated galactic cosmic rays rescued novel object recognition 3 months later in male (but not female) mice and altered cFos immunoreactivity co-activation in relevant brain regions [85]. Correlation matrices showed more strong, positive correlations in mice that received amifostine than saline-injected mice. In another study, systemic administration of 107 mg/kg of amifostine either just before or just after training in fear conditioning lowered γH2Ax in the CA1 region and increased long-term contextual fear memory in male (but not female) mice [66]. Hippocampal ΔFosB was also decreased in males and females, suggesting that the changes in hippocampus-dependent memory were a result of altered IEG expression during learning. The decrease in DSBs by amifostine may be lowering “background” DSBs—those that are occurring in the 10 s of 1000 s daily [47].

Subsequent studies have begun looking at the role of DSBs in other stages of the learning and memory process. Increased DSBs were seen in the CA1 region of the hippocampus during fear reconsolidation [67]. The genomic location was highly specific, in the promoter region for *Npas4*, but not *cFos*. Moreover, inhibition of topoIIβ in the CA1 using siRNA during the retrieval trial impaired memory and decreased DSBs on the *Npas4* promoter. Relatedly, exposing mice to whole-body gamma radiation after contextual fear conditioning increased fear memory on the subsequent day and decreased the number of cFos immunoreactive positive cells in the infralimbic cortex [68]. Thus, timing and specificity of DSBs appear to influence the effects on learning and memory. The number of parvalbumin-positive cells was also decreased in mice exposed to post-training gamma radiation [68], highlighting the importance of the cellular-subtype, which are starting to be explored.

### 4.3. Cellular Sub-Types with Adaptive DSBs

One study found that DSBs induced by contextual fear conditioning occur in neuronal and non-neuronal cells in the hippocampus and medial prefrontal cortex [69]. In neurons, genes related to synaptic function are upregulated, including IEGs such as *Arc, Npas4*, *Fos*, *Nr4a1*, *Actb*, *Ntrk2*, *Egr1,* and *Plk2*. *Arc* is also upregulated in non-neuronal cells. Glial cells—including astrocytes, oligodendrocytes, and microglia—show increased DSBs and gene expression following exposure to corticosterone. These upregulated genes are primarily related to cellular homeostasis, cell motility and adhesion, and proliferation and cell death, again indicating the specificity of DSBs for the type of stimulus [69]. These data suggest that DSBs contribute to gene expression across all major cell-types in the brain.

## 5. Double-Strand Breaks and Aging

DSB repair declines steadily with age, while DNA damage increases with age [86,87]. Age is the highest predictive risk factor for developing age-associated cognitive decline (ACD), MCI, and AD [88]. In healthy aging, hippocampus-dependent learning and memory and hippocampus volume decrease [89]. Age and disease synergistically increase the amount of pathological DSBs (i.e., increased ROS and decreased repair, causing more DSBs) [86,90,91,92]. From cellular models to post-mortem brain tissue, there is an increase in DSB markers and a decrease in DSB repair [93]. γH2Ax is increased in neurons and glia in the hippocampus of AD patients, while repair markers MRE11, RAD50, and BRCA1 are decreased. 5xFAD mice also show increased γH2Ax in hippocampal cells and decreased RAD50 mRNA, and CHO7PA2 cells have increased DNA damage measured by the comet assay [93].

The imbalance in the number of DSBs and decline in DSB repair is important to consider in the context of aging and disease. Timely DSB repair is essential in preventing apoptosis and thus is an adaptive role of DSBs in the brain. DSB repair markers remain high in *drosophila* and mice deprived of sleep after exposure to a novel environment or whole-body gamma radiation [70]. Conversely, flies or mice allowed to sleep following stimulation display lower levels of DSBs. Males are more affected by sleep deprivation than females: γH2Ax markers are higher in the frontal cortex of sleep-deprived male mice 3 and 7 h after whole-body irradiation, but not in females. Sleep is important for learning and memory processes, and there is a known circadian rhythm in IEG expression [94,95,96]. Sleep deprivation impairs learning and memory, with hippocampus-dependent learning and memory being particularly susceptible to the negative effects of sleep deprivation [97]. Sleep changes over aging and in disease states, where slow-wave and REM sleep steadily decline [98]. The importance of sleep, then, for DSBs is logical. More research is needed to understand the underlying mechanisms.

Several dominant familial AD mutations have been identified in amyloid precursor protein (APP), presenilin 1 (PS1), and presenilin 2 (PS2) genes. Transgenic mice expressing mutated human APP have higher levels of DSBs at baseline compared to WT mice, and these levels do not return to baseline levels 24 h after exploring a novel environment [64]. Moreover, APP/PS1 mice show higher DSBs than WT mice and a decrease in DSB repair signals in the hippocampus [99]. Consistent with these mouse studies, levels of γH2Ax and 53BP1 are higher in cortical areas of patients with MCI and AD compared to controls [100]. A recent genome-wide association study identified a link between alterations in the O^6^-Methylguanine-DNA Methyltransferase (MGMT) gene and AD in women [101]. MGMT is important for the DSB repair process, with decreased MGMT function linked to increased cancer risk [102]. Thus, elevated levels of pathological DSBs could be a driving factor in development of dementia or other learning- and memory-related disorders.

Behind age and biological sex, apolipoprotein E (apoE) isoform is the largest risk factor for developing late onset AD [88,103,104,105]. There are three human isoforms of apoE: E2, E3, and E4. Compared to E3 carriers, E4 carriers have a relatively higher risk of developing late onset AD, while E2 carriers are relatively protected [103]. ApoE can directly function as a transcription factor by high affinity binding double-stranded DNA [106]. E4 binds to promoter regions for genes related to inflammation (such as interleukin-6 and -8) more than E3. Conversely, E2 is associated with poor outcomes in melanoma, whereas E4 confers better outcomes (reduced tumor size, metastasis, and increased survival) [107]. Differential formation and/or repair of DSBs by distinct apoE isoforms might contribute to their differential disease risk.

In addition to AD, recent reports have implicated dysregulation of DSBs in Parkinson’s disease (PD) pathology. The phosphorylated form of alpha-Synuclein (aSyn) is the primary component of Lewy bodies. The aggregation of Lewy bodies sequesters aSyn away from the nucleus of the cell, potentially contributing to cell death [108]. Research into the typical function of aSyn has revealed that aSyn binds to DNA and that both aSyn and Tau (implicated in AD) change DNA conformation [109,110]. More recently, aSyn was shown to colocalize with γH2Ax both in vitro and in vivo, suggesting that aSyn plays a role in the DNA repair process itself. Moreover, the phosphorylation of aSyn leads to a decreased ability of aSyn to bend DNA, implicating this loss-of-function as a contributor to disease progression [111].

## 6. Conclusions

There is a growing body of literature indicating that DSBs are involved in learning and memory via the regulation of IEG expression. These pieces of evidence include the observation of transient increases in DSBs in relevant brain regions and genomic locations; impairments in memory and alterations in IEG expression following the manipulation of both DSB induction and repair; increases in DSBs in the aging and disease states; and discoveries that proteins implicated in neurodegeneration directly bind DNA and contribute to DSB repair. At first glance, it seems counterintuitive that if DSBs are involved in learning and memory that inducing them pharmacologically would impair memory. One possible reason is that the increase in DSBs in the absence of a behavioral experience might be problematic if it would prevent an increase in DSB in response to a behavioral experience. There might be hot spots for DSBs that are triggered by both pharmacological stimulation and behavioral experiences and DSBs by themselves might not be sufficient to generate memories. With regard to the enhanced contextual fear memory following post-training irradiation, DSBs in spots primed by the behavioral experience might be further enhanced by radiation exposure. It is also possible that part of the memory signal relates to the repair of DSBs. More research is needed to clarify the nuances of this precarious mechanism, as precision appears to be key in distinguishing harmful from adaptive DSBs. The pathological:adaptive ratio may be key in understanding when intervention is necessary and how to best intervene to prevent dysregulation and subsequent disease.

Looking towards future studies, one possible major advancement lies in methodology. The majority of studies have used γH2Ax to identify the cellular and genomic location of DSBs, yet γH2Ax is an indirect marker. Labeling DSBs themselves, without relying on secondary markers, will be an important step in understanding the function of transient, stimulus-induced DSBs: indeed, there are groups working on methods to tag DSBs, such as Breaks Labeling In Situ and Sequencing (BLISS) [112], and END-seq [113,114]. Moreover, there is a need for more precise tools to manipulate DSBs to fully understand the effects of interfering with typical DSB formation and repair on learning and memory. For example, the ability to inhibit DSB formation specifically on IEGs (such as *c-fos* or *Arc*) would determine the necessity of DSBs for the expression of these genes. There is a wealth of opportunity to continue exploring adaptive DSBs, filling in missing pieces of the dynamic process of synaptic plasticity.
